# The Cornwall Helicopter

**Published:** 1991-12

**Authors:** N. Sellwood

**Affiliations:** Consultant in Accident and Emergency Royal Cornwall Hospital Truro


### Letter to the Editor

Dear Sir

**Article "Effect of the Cornwall Helicopter Ambulance on Ambulance Service Emergency Response Time" by Andrew Rouse, MFPHM**

Dr. Rouse's article unfortunately severely distorts the facts about the use of the First Air Ambulance and misreports attitudes of professionals and others in Cornwall. Whilst his statistical analysis of emergency response times may be accurate, it must be considered doubtful that the extrapolations stated are valid. The article, although correctly itemising the advantages of the Air Ambulance, then analyses just one parameter and ignores the rest.

The reasons for the introduction of the First Air Ambulance in Cornwall may be obscure to Dr. Rouse but the decision was made after years of frustration caused by the geographical shape of Cornwall, the wide scatter of its population, the sporadic and haphazard location of emergencies and the congestion of the roads. No road based fleet of ambulances can deliver the same level of care without the support from the air, unless the County be flooded with paramedic crewed ambulances. This is both expensive and inefficient in terms of crew usage.

Emergencies are not few and far between in Cornwall: there are as many emergencies as in other Health Districts of equivalent size. In the year 16th October 1990 to 15th October 1991, 380 patients were evaluated in the Resuscitation Room of the Accident and Emergency Department at Truro, of which 180 were trauma cases. This is not to mention the numerous patients with emergency conditions at home who are admitted directly to the wards, often transported by First Air.

Muddled thinking seems to take over later in the article when he discusses the need for a helicopter in Cornwall. If there were 150 trauma deaths in residents of Cornwall in 1988 (when the Air Ambulance was operational) this figure may have been higher were it not for this valuable service. There would have been more deaths than this considering the influx of 3 million visitors in the year. It also does not account for the medical emergencies handled by crews in First Air.

The unpalatability of putting a cost on a death is understandable but it is undertaken by the Department of Transport quite routinely. They have calculated the cost to the State of a trauma death as £264,881 in 1988. If the Air Ambulance cost is £44,000 per life saved, it must be good value for money.

The reality is that none of this figure crunching tells us how to manage individual cases as and when they present. There are plenty of anecdotes illustrating the effective use of First Air in life saving situations and there are quite a few people in Cornwall who know they owe their lives and health to it. I think better advice to Health Authorities considering the introduction of a helicopter service would be to enquire of the professionals involved in the day to day running of First Air, of those who treat the patients in it and of the clinicians receiving the patients transported by it. No patient's treatment should be calculated in terms of just one of the many and varied components which go to make up the whole.

Yours faithfully,
Mr. N. Sellwood, FRCS
Consultant in Accident and Emergency
Royal Cornwall Hospital
Truro

